# Associations of Antidepressants With Atrial Fibrillation and Ventricular Arrhythmias: A Systematic Review and Meta-Analysis

**DOI:** 10.3389/fcvm.2022.840452

**Published:** 2022-03-25

**Authors:** Yalin Cao, Mingyu Zhou, Huaiyun Guo, Wengen Zhu

**Affiliations:** ^1^Department of Cardiology, Guizhou Provincial People's Hospital, Guiyang, China; ^2^Second Clinical Medical College, Nanchang University, Nanchang, China; ^3^College of Pharmacy, Nanchang University, Nanchang, China; ^4^Department of Cardiology, The First Affiliated Hospital of Sun Yat-sen University, Guangzhou, China

**Keywords:** antidepressants, arrhythmia, atrial fibrillation, sudden cardiac death, meta-analysis

## Abstract

**Background:**

Several published studies have disagreements on whether the use of antidepressants is associated with increased risk of arrhythmias. In this study, we performed this meta-analysis to assess the association of antidepressants with cardiac arrhythmias in patients who require antidepressants.

**Methods:**

The PubMed and Embase databases were systematically searched until December 2021 to find studies that investigated the association between antidepressant use and cardiac arrhythmias. Studies that assessed the effects of any antidepressant on arrhythmias in patients who require antidepressants compared with those who require no antidepressants were included. We used a random-effects model to pool the adjusted risk ratios (RRs) and 95% confidence intervals (CIs). The stability of the results was examined by omitting an individual study at a time.

**Results:**

A total of 3,396 studies were screened and 6 studies with 2,626,746 participants were finally included in this meta-analysis. When compared with no antidepressants, the use of antidepressants was significantly associated with an increased risk of atrial fibrillation (RR = 1.37, 95% CI: 1.16–1.61). However, there was no difference in the risk of ventricular arrhythmias or sudden cardiac death (RR = 1.33, 95% CI: 0.88–2.01) between the two studied groups. In the subgroup analysis, tricyclic antidepressants (RR = 1.12, 95% CI: 0.89–1.41), selective serotonin reuptake inhibitors (RR = 1.46, 95% CI: 0.63–3.38), and selective serotonin reuptake inhibitors (RR = 0.99, 95% CI: 0.97–1.01) did not increase the risk of ventricular arrhythmias and/or sudden cardiac death.

**Conclusion:**

Recently published data suggested that the use of antidepressants did not increase the risk of ventricular arrhythmias or sudden cardiac death. Antidepressants were associated with an increased risk of atrial fibrillation but that still needs further confirmation.

## Introduction

A published study of the United States with 36,309 respondents demonstrated that 10.4% of respondents suffered from a 12-month depressive disorder, and 20.6% of respondents suffered from a lifetime depressive disorder (1). Previously published studies have indicated that depression might increase the risks of chronic diseases (2). As such, the use of antidepressants is not rare in the management of major depressive disorders (3). Tricyclic antidepressants (TCAs) and selective serotonin reuptake inhibitors (SSRIs) are the most frequently used, whereas other antidepressants include serotonin-norepinephrine reuptake inhibitors (SNRIs), mono-amino oxidase inhibitors (MAOs), specific serotonergic antidepressants (NaSSAs), and serotonin antagonist and reuptake inhibitors (SAIRs). However, antidepressant medications sometimes have no effects on treatment-resistant patients and even lead to unexpected adverse cardiovascular outcomes (4, 5).

Arrhythmia is a widely distributed disease that needs better medical resources and can lead to life and economic burden (6). Atrial fibrillation (AF) is the most frequent type of arrhythmias. Ventricular arrhythmia or sudden cardiac death (VA/SCD) are also severe outcomes associated with fatality. Several studies have investigated the association between antidepressant use and the risks of AF or VA/SCD, but their results remain controversial. The study of Garg et al. suggested that the treatment of antidepressants has a weaker association with incident AF, which might reflect severe depressive symptoms but not the limited effectiveness of drugs (7). However, the study of Rayne et al. suggested that a low dose of TCAs did not increase the risk of SCD (8), and some other studies reported that there was no reliable relationship between antidepressants and arrhythmias, including cAF, SCD, and VA (9–11). Therefore, the effect of antidepressants on arrhythmia risks remains uncertain. To assess the risk of arrhythmias in patients who require antidepressants, we performed a meta-analysis to assess the association between antidepressant use and the risks of AF or VA/SCD.

## Methods

We used the Preferred Reporting Items for Systematic Review and Meta-Analyses (PRISMA) 2020 statement to report our findings (12). Ethical approval was not required since our meta-analysis was based on the literature that was already published. The data that support the findings of this meta-analysis would be available from the corresponding authors on reasonable requests.

### Data Sources and Search Strategy

We comprehensively searched the PubMed and Embase databases from inception to December 2021 for the relevant studies that reported whether antidepressants increased the risks of arrhythmias. According to our search strategies, two kinds of search terms, namely, antidepressants (e.g., antidepressants, TCAs, SSRIs, SNRIs, and NaSSAs) and arrhythmias (e.g., AF, atrial flutter, and VA), were included. Our concrete search strategies of the PubMed and Embase databases are mentioned in Supplementary Table 1. No language restrictions were used in our search.

### Eligibility Criteria

We included studies fulfilling the following criteria:

(1) Studies assessed the effects of any antidepressant on arrhythmias in patients taking antidepressants. The comparison population was those not taking antidepressants.
(2) The outcomes were arrhythmias including AF or VA/SCD.
(3) The effects of antidepressant use on the studied outcomes were expressed as adjusted effect estimates risk.

We excluded studies having the following criteria:

(1) Studies with unadjusted data were excluded.
(2) Comparisons between different antidepressants were excluded.
(3) Study types such as reviews, case reports, editorials, letters, and meeting abstracts were excluded.

In the case of multiple publications using the same data, the study with the longest follow-up time or with the largest number of participants was included.

### Data Extraction

According to the process of study selection, two authors independently screened the studies based on titles and abstracts. Then, the full texts were assessed in detail based on the inclusion criteria to identify the final eligibility. All disagreements were resolved by discussion. Two authors also extracted data independently; the first author, publication year, data sources, country, sample size, mean age, sex, follow-up time, interventions, antidepressants in the control groups, adjusted risk ratios (RRs), and 95% confidence intervals (CIs) were included.

### Outcomes

Our main outcomes were AF and VA/SCD. VA/SCD was defined as ventricular tachycardia, torsades de pointes, ventricular fibrillation, ventricular flutter, sudden cardiac arrest, and SCD. Since SCD was specific and often caused by VA, we set VA and SCD as a composite group.

### Study Quality Assessment

According to the eligibility, the Newcastle-Ottawa Scale (NOS) was used to evaluate the quality of observational studies, with a total score of 9 points. The NOS of cohort studies included the selection of cohorts (i.e., 0–4 points), the comparability of cohorts (i.e., 0–2 points), and the assessment of outcomes (i.e., 0–3 points). A quota was defined to evaluate the concrete quality, that is, the total of scores > 6 was considered moderate to high quality, while the total of scores < 6 was regarded as low quality (13).

### Data Analysis

All the statistical analyses were performed using the Review Manager version 5.4 software (the Cochrane Collaboration 2014, Nordic Cochrane Center Copenhagen, Denmark; https://community.cochrane.org/). The *I*^2^ statistic was used to assess the statistical heterogeneity. If **I**^2^ values were ≤25%, 50%, and ≥75%, the included studies will correspond to low, moderate, and high heterogeneity, respectively. We extracted odds ratios from the case-control studies and hazard ratios from cohort studies. Ultimately, we selected RRs and 95% CIs to represent all the effect estimates in the results, which did not influence the final interpretation (14). The effects of antidepressant use on the studied outcomes were expressed as adjusted RRs and 95% CIs. We calculated the adjusted RRs and 95% CIs to their corresponding natural logarithms and standard errors. To better evaluate studies with high heterogeneity, we applied a random-effects model to pool our data. In the sensitivity analysis, we examined the stability of the results after omitting an individual study at a time. We also reperformed the analysis using a fixed-effects model. In addition, a subgroup analysis based on the drug classes of antidepressants was performed. We did not assess the publication bias because the number of included studies was < 10. In this study, a *p* < 0.05 indicated statistical significance.

## Results

### Study Selection

**Figure 1:**
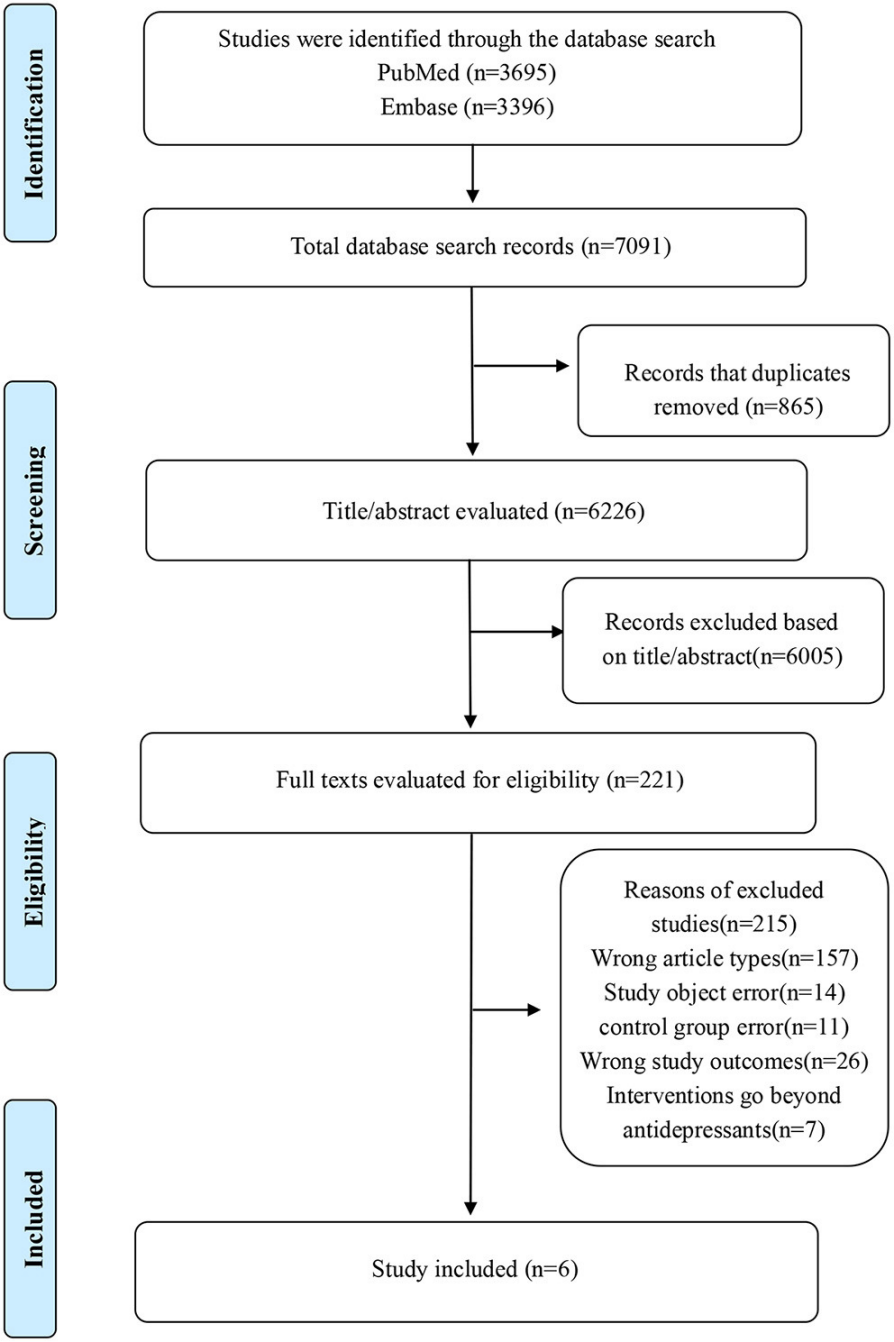
The concrete retrieval process flowchart of our meta-analysis.

In general, 7,091 records were searched from PubMed and Embase databases. After screening all the titles and abstracts, 6,005 records were excluded. Then, 215 records were excluded based on the full texts. Ultimately, 6 studies [6 cohort studies (7, 8, 15–18)] with 2,626,746 participants were included. The retrieval process flowchart is shown in Figure 1.

The detailed baseline characteristics of the included studies are presented in Table 1. There are 5 studies from the United States and 1 study from Denmark, and there are no duplicates in the data sources. Many antidepressant drug classes were estimated, including TCAs, SSRIs, and SNRIs. The definition of outcomes in our included studies is displayed in Supplementary Tables 2, 3. The detailed confounding factors of included studies are presented in Supplementary Table 4.

**Table 1:** Baseline characteristics of the included studies.

**Study**	**Country**	**Data source**	**Study design**	**Females (%)**	**Mean age (y)**	**Follow-up time (y)**	**Participants (*n*)**	**Intervention antidepressants**	**The number of confounders**	**Confounder factors**
Fenger-Gron et al. (15)	Denmark	All Danes initiating antidepressant treatment from 2000 to 2013	Cohort study	59.2	N/A	N/A	785,254	SSRIs (citalopram, escitalopram), NaSSAs (Mirtazapine, Venlafaxine)	18	Marital status, age, sex, diabetes, ischaemic heart disease, dyslipidemia, hypertension, heart failure, stroke, peripheral artery disease, anemia, thyroid disorder, chronic kidney disease, schizophrenia or schizoaffective disorder, bipolar affective disorder dementia, alcohol abuse and/or other substance abuse.
Fung et al. (16)	USA	Virtual Research Data Center	Cohort Study	58.5	65.0	3.7	1,265,921	SSRIs (Citalopram, Escitalopram), SNRIs	6	Gender, race, degree of low-income subsidy, rural residence indicator, and year of Part D entry, CCW comorbidity flags
Garg et al. (17)	USA	Multi-Ethnic of Atherosclerosis	Cohort Study	53	62	12.9	6,664	TCAs, MAOs, and other nontricyclic antidepressants	15	Age, sex, race, education, income, clinic site, cigarette smoking, body mass index, height, diabetes mellitus, glucose, systolic blood pressure, moderate and vigorous physical activity, statin use, antihypertensive use, and current alcohol use.
Garg et al. (7)	USA	Atherosclerosis Risk in Communities	Cohort Study	49	58.8	23.4	11,445	SSRIs, TCAs MAOs	5	Age, sex, race-center, education, height
Ray et al. (8)	USA	Tennessee Medicaid	Cohort Study	77.1	46.5	5	481,744	TCAs, SSRIs	10	Calendar year, demographic characteristics (age, sex, race), measures of medical care utilization, comorbidity, identified from medical care encounters in the preceding 365 days, frequency of outpatient encounters, antipsychotic use, mental illness, serious noncardiovascular somatic illness, cardiovascular disease.
Whang et al. (18)	USA	Nurses' Health Study	Cohort Study	100	56.7	28	75,718	SSRIs (sertraline, fluoxetine, paroxetine, citalopram), TCAs (amitriptyline, paroxetine, nortriptyline)	16	Age, beginning year of follow-up, smoking status, body mass index, alcohol intake, menopausal status and postmenopausal hormone use, usual aspirin use, multivitamin use, vitamin E supplement use, hypercholesterolemia, family history, history of stroke, n-3-fatty acid intake, alpha linolenic acid intake, moderate/vigorous physical activity, CHD, hypertension and diabetes.

*TCAs, tricyclic antidepressants; SSRIs, selective serotonin reuptake inhibitors; SNRIs, serotonin-norepinephrine reuptake inhibitors; MAOs, mono-amino oxidase inhibitors; NaSSAs, specific serotonergic antidepressants; CCW, Chronic Conditions Warehouse; CHD, coronary heart disease*.

### AF Events Related to Antidepressant Use

**Figure 2:**
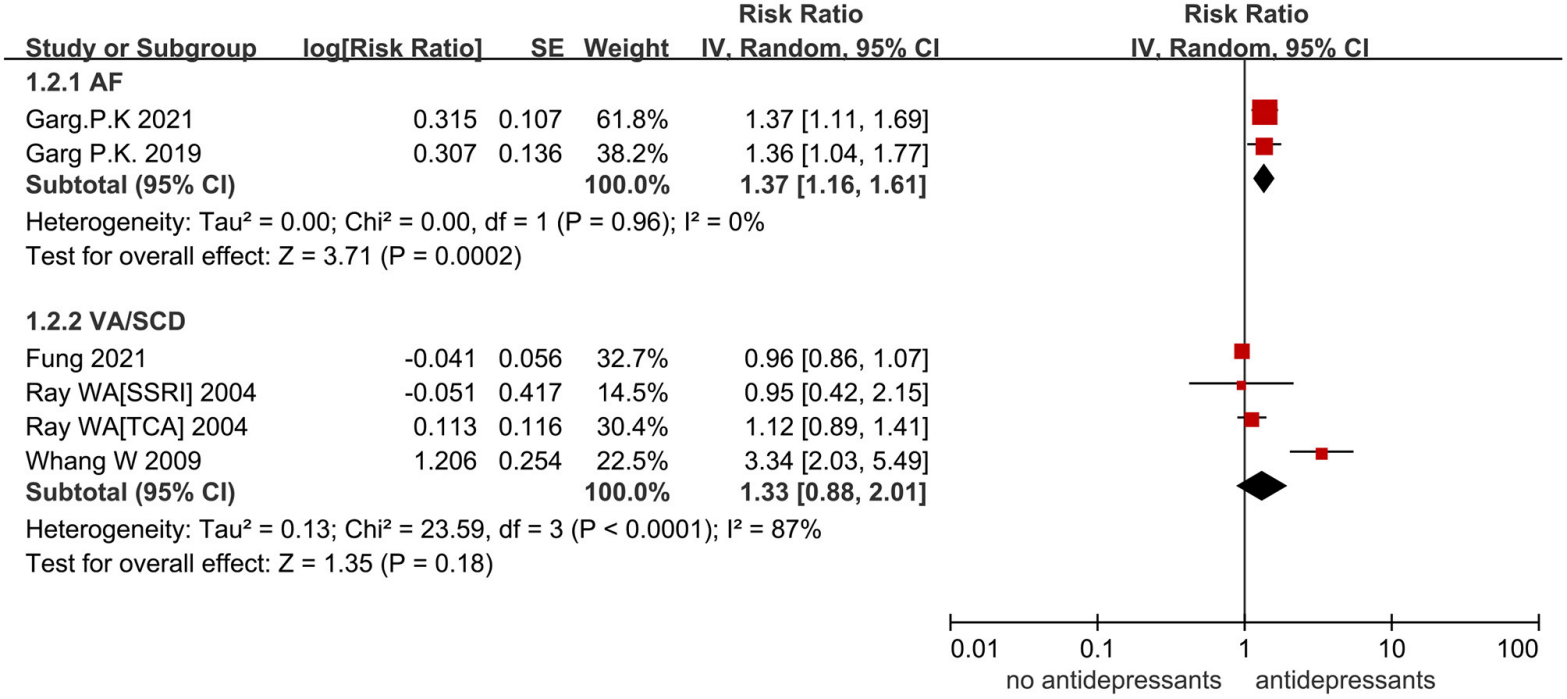
A forest plot of the association between antidepressant use and the increased risk of arrhythmias compared with nonusers. AF, atrial fibrillation; VA, ventricular arrhythmia; SCD, sudden cardiac death; RR, risk ratio; TCA, tricyclic antidepressant; SSRI, selective serotonin reuptake inhibitor; SNRI, serotonin norepinephrine reuptake inhibitor; CI, confidence interval; SE, standard error; IV, inverse of the variance.

Among the 3 studies that reported the outcome of AF, the study of Garg et al. (7) suggested the association between antidepressants and incident AF in a cohort of middle and old-aged adults. Garg et al. (17) reported the increased risk of AF that is associated with depression symptoms. The risk of AF associated with the use of antidepressants seemed more pronounced in women instead of men. Fenger-Grøn et al. (15) indicated the incidence rate of AF in different periods after using antidepressants. In the pooled analysis, antidepressant use did not increase the risk of AF in the comparison with no antidepressants (RR = 1.82, 95% CI: 0.94–3.55, *P* = 0.08, *I*^2^ = 98%) (Supplementary Figure 1). After omitting the study of Fenger-Grøn et al., the heterogeneity decreased significantly from 98% to 0%. Thus, we reperformed the analysis by removing this study and found that the use of antidepressants significantly increased the risk of AF (RR = 1.37, 95% CI: 1.16–1.61, *P* = 0.0002, *I*^2^ = 0%; Figure 2).

### VA/SCD Events Related to Antidepressant Use

A total of 3 studies concentrated on whether antidepressants increased the risk of VA/SCD. The study of Whang et al. (18) indicated an elevation in the risk of SCD in a women cohort who reported antidepressant use. The study of Fung et al. (16) demonstrated the reduced risk of both citalopram and escitalopram with cumulative use of more than 12 months through medical data of old adults. In the pooled analysis, the use of antidepressants had no association with the increased risk of VA/SCD (RR = 1.33, 95% CI: 0.88–2.01, *I*^2^ = 87%; *P* = 0.18) (Figure 2). For heterogeneity analysis, an *I*^2^ of 87% signified high heterogeneity. After omitting the study of Whang et al., *I*^2^ decreased from 87 to 0%

### Subgroups Analysis

**Figure 3:**
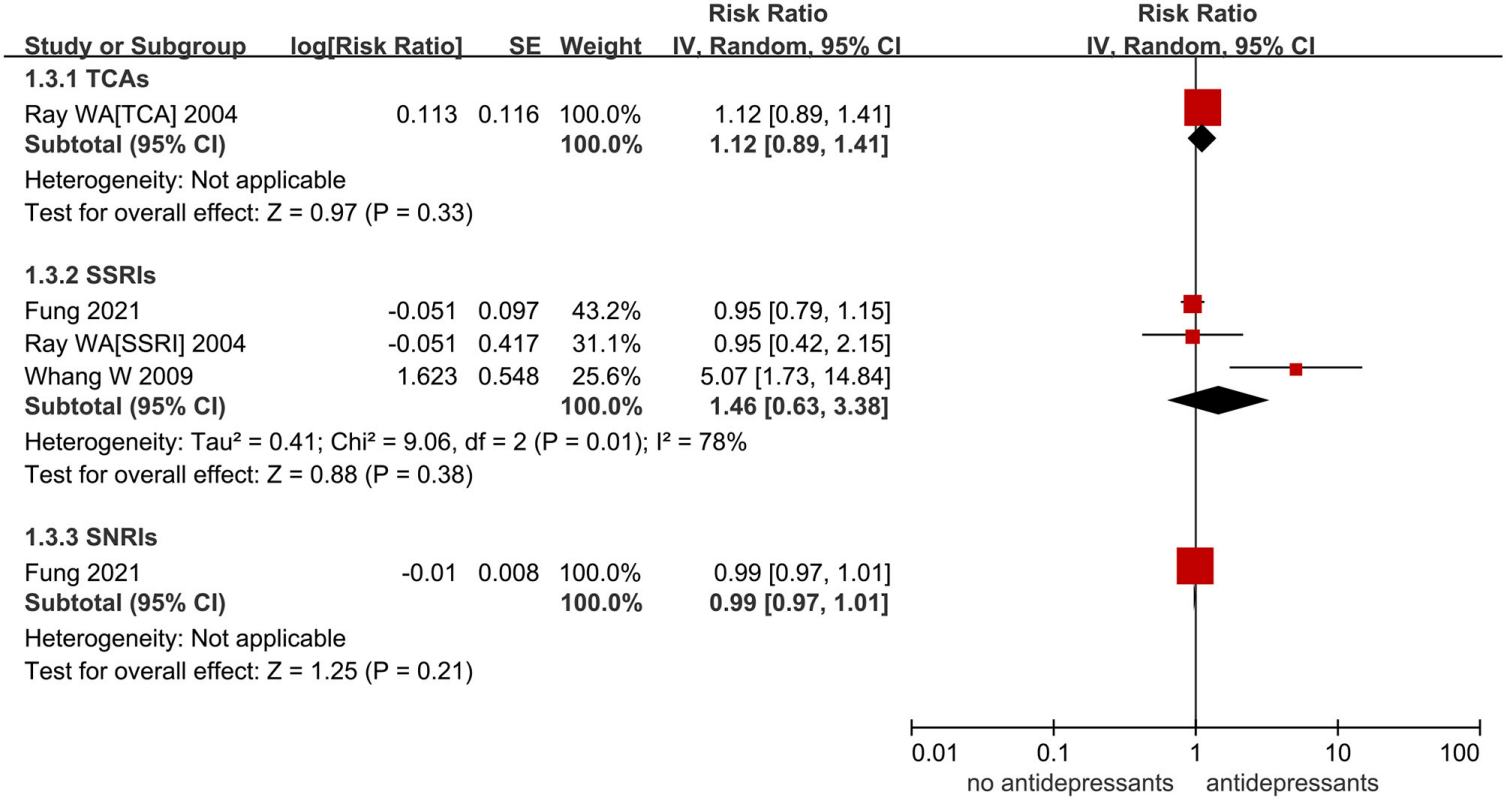
A forest plot of the use of antidepressant on ventricular arrhythmia (VA)/sudden cardiac death (SCD) in patients who require antidepressants based on the classes of antidepressants, including TCAs, SSRIs, and SNRIs. TCAs, tricyclic antidepressants; SSRIs, selective serotonin reuptake inhibitors; SNRIs, serotonin-norepinephrine reuptake inhibitors; RR, risk ratio; CI, confidence interval; SE, standard error; IV, inverse of the variance.

The subgroup analysis based on drug classes of antidepressants was not performed for the outcome of AF due to the limitation of data. For the risk of VA/SCD, studies were divided into 3 subgroups based on the types of antidepressants. As shown in Figure 3, TCAs (RR = 1.12, 95% CI: 0.89–1.41, *P* = 0.33), SSRIs (RR = 1.46, 95% CI: 0.63–3.38, *P* = 0.38), and SNRIs (RR = 0.99, 95% CI: 0.97–1.01, *P* = 0.21) did not increase the risk of VA/SCD.

### Sensitivity Analysis

In the analysis of AF, after removing the study of Fenger-Grøn et al. (15), the heterogeneity decreased significantly from 98 to 0%. The reason might contribute to a distinct difference in the follow-up time between the studies of Fenger-Grøn et al. and Garg et al. In the analysis of VA/SCD, omitting the study of Whang et al. (18) also decreased the *I*^2^ from 87 to 0%. It might be because it contained a women cohort, which may influence the result by sex. The outcomes of SCD in this study may not be caused by the risk of arrhythmia precisely. Omitting the aforementioned studies, the origin results remained stable. When we transformed the analysis to the fixed-effects models, the results of the association between antidepressants and the risk of VA/SCD changed slightly.

### Quality Assessment

After assessing their quality based on the NOS score, the scores of included observational studies were all between 6 and 8, which represented moderate to high quality (Supplementary Table 5).

## Discussion

Although the presented meta-analysis is based on the current published literature, we took two outcomes to represent arrhythmias, namely, AF and VA/SCD. Overall, the use of antidepressants did not increase the risks of VA/SCD. Antidepressants were associated with an increased risk of AF.

For the association between antidepressants and the risk of AF, because of the limitation that most studies concentrated on VAs, the potential mechanisms between antidepressants and AF have no accurate interpretation, and we postulated the following hypothesis. Primarily, several previous studies demonstrated that the reducing effect of 5-hydroxytryptamine (5-HT) on L-type Ca^2+^ current (I_CaL_) and action potential duration at 50% (APD_50_) seemed to have a relationship with AF (19–21). Moreover, the increased risk may be because of the indication for antidepressant use instead of actual antidepressant use (22). In the data from the Danish study that adjusted for sociodemographic and clinical confounds, there was a significantly increased risk of AF in antidepressant users during days 1–15 and days 16–30 before antidepressant initiation. Since antidepressants could not lead to AF before the initiation, the indication for the treatment of antidepressants or common characteristics of patients could reasonably be suspected to be the cause. Some prior studies reported that positive association between depression and AF since depression might increase the activation of the autonomic nervous system, the hypothalamic–pituitary–adrenal axis, and the renin-angiotensin aldosterone system. Owing to different psychological assessment methods that could lead to different results, investigating the effect of depression in AF was challenging (15, 23). Simultaneously, some interpretations suggested that enhanced AF symptoms might be due to lateralization of cerebral activity caused by emotional stress and high sympathetic nervous activity caused by major depressive disorder (24, 25).

Compared with no antidepressants, our analysis indicated that the use of TCAs, SSRIs, and SNRIs did not increase the risk of VA/SCD. In the 3 included studies of VA/SCD, only the study of Fung et al. (16) adopted the outcome that was completely consistent with our meta-analysis (composite VA/SCD). The outcomes of the other two studies focused more on SCD. Furthermore, only the study of Fung et al. (16) indicated that the cumulative time of antidepressant use might have an effect on the risk of VA/SCD. Patients who took cumulative use of > 12 months had a lower risk of arrhythmias. Since there were no definite reasons for this phenomenon, it was possible that the risk of arrhythmias increased significantly in the beginning, so the treatment of antidepressants was stopped. Immortal time bias should also be taken into account. A previously published meta-analysis reported that antidepressants had no valid effect on the increased risk of SCD in the comparison with different antidepressants (26). The concrete reasons are still discussed, and we postulated the following hypothesis. As for TCAs, TCAs can inhibit the sodium channel conductance and delay 0-phase cardiac depolarization, which can lead to the slower conduction velocity of His-Purkinje fibers and ventricular myocardium, due to which the prolongation of the Q wave, R wave, and S wave (QRS) complex happens on ECG (24). For SSRIs, fluoxetine and citalopram can inhibit the human ether-a-go-go-related gene (hERG) potassium channel that plays an important role in the repolarization of the cardiac action potential, which may have a relationship with the prolongation of the corrected QT interval (QTc) interval and lead to adverse reactions, such as VAs (28, 29).

Several studies reported that using TCAs had a lower risk of VA/SCD than SSRIs (30) because a moderate to a high dose of TCA use seemed to increase the risk of VA/SCD instead of a low dose. However, the use of TCAs was mainly in the case of low doses based on obvious side effects (31). As for SSRIs, several studies reported with greater evidence that the prolongation of the QTc interval and cardiotoxicity was associated with antidepressant overdose, whereas evidence rarely existed when treating with therapeutic administration (27, 32). However, we did not analyze the relationship between drug dose and arrhythmias because the data on dose and types of antidepressants varied among the included studies, which was limited and beyond the scope of discussion. Despite SNRIs being considered as the newer classes of antidepressants that were not fully explored, several studies indicated that they seemed not to increase the risk of arrhythmias, including SCD and VA (10, 11, 33, 34).

With the increasing attention to depression, the use of antidepressants has also increased and expanded (35). In addition, a previously delivered meta-analysis demonstrated that the risk of VA was related to patients with depression, especially patients with coronary heart disease, so the treatment with antidepressants should be administered carefully (36). However, our discussion on whether antidepressants increased the risks of AF is based on current studies. Further studies can find more definite conclusions in doses and classes of antidepressants.

## Limitations

This meta-analysis has the following limitations that should be taken into account. First, there were insufficient studies in subgroups, such as AF, which might cause the worse accuracy of results. Second, since the included studies used different data sources and analysis methods, the residual confounders should be included. Third, owing to the limitation of drug doses in the included literature, the statistical analysis about the doses of antidepressants was not supported. Moreover, a comparison between different antidepressants was excluded in our meta-analysis. In sensitivity analysis, after omitting one study at a time, heterogeneity decreased significantly, such that a greater heterogeneity between studies might influence the validity of the results. Finally, further studies could focus on the relationship between doses of antidepressants and cardiac arrhythmias.

## Conclusion

According to the published literature, our analysis demonstrated that the use of antidepressants did not increase the risks of VA/SCD. Antidepressants were associated with an increased risk of AF. Further studies are required to confirm these findings.

## Data Availability Statement

The original contributions presented in the study are included in the article/Supplementary Material, further inquiries can be directed to the corresponding author/s.

## Author Contributions

All authors listed have made a substantial, direct, and intellectual contribution to the work and approved it for publication.

## Funding

This study was supported by the Clinical Research Center Project of the Department of Science and Technology of Guizhou Province [Grant No. (2017) 5405].

## Conflict of Interest

The authors declare that the research was conducted in the absence of any commercial or financial relationships that could be construed as a potential conflict of interest.

## Publisher's Note

All claims expressed in this article are solely those of the authors and do not necessarily represent those of their affiliated organizations, or those of the publisher, the editors and the reviewers. Any product that may be evaluated in this article, or claim that may be made by its manufacturer, is not guaranteed or endorsed by the publisher.
