# Supplementary material for: Associations of Antidepressants With Atrial Fibrillation and Ventricular Arrhythmias: A Systematic Review and Meta-Analysis
Source: Front Cardiovasc Med. 2022 Mar 25;9:840452. doi: 10.3389/fcvm.2022.840452 (PMC8990315; doi:10.3389/fcvm.2022.840452)
Supplement: Supplementary file 1 [file Data_Sheet_1.pdf]

Supplementary Table 1 The literature search strategies on databases

Databases	Queries	Number of studies
**PubMed**		
#1	Antidepressive Agents OR Antidepressant OR Antidepressant Drug	186858
#2	Selective Serotonin Reuptake Inhibitor OR Selective Norepinephrine Reuptake Inhibitor OR Serotonin Uptake Inhibitors OR Neurotransmitter Uptake Inhibitors OR Tricyclic Antidepressant OR Tetracyclic antidepressants OR Monoamine Oxidase Inhibitors OR Norepinephrine and specific serotonergic antidepressant	181962
#3	Agomelatine OR Bupropion OR Citalopram OR Desvenlafaxine OR Maprotiline OR Duloxetine OR Escitalopram OR Fluoxetine OR Fluvoxamine OR Milnacipran OR Mirtazapine OR Paroxetine OR Reboxetine OR Sertraline OR Venlafaxine OR Vortioxetine	44696
#4	Cardiac Arrhythmia OR Cardiac Dysrhythmia OR Sinus Arrhythmia OR Sinoatrial Arrhythmia OR Sinus Tachycardia OR Sinus bradycardia OR sinus arrhythmia OR Cardiac arrest OR Sudden Cardiac Death OR Sinoatrial Block OR Sick Sinus Syndrome OR Sinus Node Dysfunction OR bradycardia tachycardia syndrome OR atrial escape rhythm OR junctional escape rhythm	276987
#5	ventricular escape rhythm OR Premature Complex OR Atrial Extrasystole OR Atrial premature contraction OR Atrial tachycardia OR Atrial flutter OR Atrial fibrillation OR Supraventricular Tachycardia OR Ventricular Premature Complex OR Ventricular premature contraction OR Ventricular Arrhythmia OR Ventricular tachycardia OR Ventricular Flutter OR ventricular fibrillation OR sinoatrial block OR SA block OR Atrioventricular block OR AV Block OR Intraventricular block OR intra-atrial block OR atrioventricular node reentrant tachycardia OR Atrioventricular reentrant tachycardia OR Chaotic atrial tachycardia OR Wolff Parkinson White syndrome OR torsade de points	230362
	(#1 OR #2 OR #3) AND (#4 OR #5)	3695
**EMBASE**		
#1	(antidepressive AND agents OR 'antidepressant' OR antidepressant) AND 'drug'	142340
#2	'selective serotonin reuptake inhibitor' OR 'selective norepinephrine reuptake inhibitor' OR 'serotonin uptake inhibitors' OR 'neurotransmitter uptake inhibitors' OR 'tricyclic antidepressant' OR 'tetracyclic antidepressants' OR 'monoamine oxidase inhibitors' OR 'norepinephrine and specific serotonergic antidepressant'	45361
#3	agomelatine OR bupropion OR citalopram OR desvenlafaxine OR maprotiline OR duloxetine OR escitalopram OR fluoxetine OR fluvoxamine OR milnacipran OR mirtazapine OR paroxetine OR reboxetine OR sertraline OR venlafaxine OR vortioxetine	123251
#4	'cardiac arrhythmia' OR 'cardiac dysrhythmia' OR 'sinoatrial arrhythmia' OR 'sinus tachycardia' OR 'sinus bradycardia' OR 'sinus arrhythmia' OR 'cardiac arrest' OR 'sudden cardiac death' OR 'sinoatrial block' OR 'sick sinus syndrome' OR 'sinus node dysfunction' OR 'bradycardia tachycardia syndrome' OR 'atrial escape rhythm' OR 'junctional escape rhythm'	142390
#5	'ventricular escape rhythm or premature complex' OR 'atrial extrasystole' OR 'atrial premature contraction' OR 'atrial tachycardia' OR 'atrial flutter' OR 'atrial fibrillation' OR 'supraventricular tachycardia' OR 'ventricular premature complex' OR 'ventricular premature contraction' OR 'ventricular arrhythmia' OR 'ventricular tachycardia' OR 'ventricular flutter' OR 'ventricular fibrillation' OR 'sinoatrial block' OR 'sa block' OR 'atrioventricular block' OR 'av block' OR 'intraventricular block' OR 'intra-atrial block' OR 'atrioventricular node reentrant tachycardia' OR 'atrioventricular reentrant tachycardia' OR 'chaotic atrial tachycardia' OR 'wolff parkinson white syndrome' OR 'torsade de points'	300835
#6	#1 OR #2 OR #3	225275
#7	#4 OR #5	403807
#8	#6 AND #7	3396

Supplementary Table 2. The assessment of AF in our included literature

Included literature	the assessment of AF	Types of AF
Garg, P. K 2021	ICD-9-CM (427.31, 427.32)	Incident AF
Garg PK 2019	ECGs or ICD-9-CM (427.31, 427.32) or medical record	Incident AF
Fenger-Gron M 2019	ICD-8(427.93, 427.94) or ICD-10 (I48)	Not available

ICD= International Classification of Diseases; ECG= Electrocardiogram; AF=atrial fibrillation

Supplementary Table 3. The assessment of VA/SCD in our included literature

Included literature	the assessment of VA/SCD
Fung, K. W.2021	ICD-9-CM/ICD-10-CM(427.1/I47.2, 427.41/I49.01, 427.42/I49.02, 427.5/I46.9, 798.1)
Whang W 2009	medical records or autopsy findings
Ray WA 2004	death certificates and medical records

Supplementary Table 4. The detailed confounding factors in our included literature

Included literature	detailed confounding factors
Garg, P. K 2021	Age, sex, race-center, education, height
Fung, K. W.2021	Gender, race, degree of low-income subsidy, rural residence indicator, and year of Part D entry, CCW comorbidity flags
Whang W 2009	Age, beginning year of follow-up, smoking status, body mass index, alcohol intake, menopausal status and postmenopausal hormone use, usual aspirin use, multivitamin use, vitamin E supplement use, hypercholesterolemia, family history, history of stroke, n-3-fatty acid intake, alpha linolenic acid intake, moderate/vigorous physical activity, CHD, hypertension and diabetes.
Garg PK 2019	Age, sex, race, education, income, clinic site, cigarette smoking, body mass index, height, diabetes mellitus, glucose, systolic blood pressure, moderate and vigorous physical activity, statin use, antihypertensive use, and current alcohol use.
Fenger-Gron 2019	Marital status, age, sex, diabetes, ischaemic heart disease, dyslipidaemia, hypertension, heart failure, stroke, peripheral artery disease, anaemia, thyroid disorder, chronic kidney disease, schizophrenia or schizoaffective disorder, bipolar affective disorder dementia, alcohol abuse and/or other substance abuse.
Ray WA 2004	Calendar year, demographic characteristics (age, sex, race), measures of medical care utilization, comorbidity, identified from medical care encounters in the preceding 365 days, frequency of outpatient encounters, antipsychotic use, mental illness, serious noncardiovascular somatic illness, cardiovascular disease.

Abbreviations: CCW= chronic condition warehouse; CHD= coronary heart disease;

Supplementary Table 5. Study quality assessment of the cohort studies based on the NOS scale

Author	Selection				Comparability	Outcome			Total scores
	Representativeness of the exposed cohort	Selection of the non-exposed cohort	Ascertainment of exposure	Demonstration that outcome of interest was not present at the start of study	Comparability of cohorts on the basis of the design or analysis	Assessment of outcome	Was follow-up long enough for outcomes to occur	Adequacy of follow up of cohorts	
Garg, P. K 2021	1	1	1	1	2	1	1	1	8
Fung, K. W.2021	1	1	1	0	2	1	0	1	8
Whang W 2009	0	1	1	1	2	0	1	1	7
Garg PK 2019	1	1	1	1	2	1	0	1	8
Fenger-Gron 2019	1	1	1	1	2	1	1	0	8
Ray WA 2004	1	1	1	0	2	1	1	1	8

NOS= Newcastle-Ottawa score

Study or Subgroup	log[Risk Ratio]	SE	Weight	Risk Ratio IV, Random, 95% CI
**1.1.1 AF**				
Fenger-Gron 2019	1.157	0.033	34.2%	3.18 [2.98, 3.39]
Garg.P.K 2021	0.315	0.107	33.2%	1.37 [1.11, 1.69]
Garg P.K. 2019	0.307	0.136	32.6%	1.36 [1.04, 1.77]
**Subtotal (95% CI)**			**100.0%**	**1.82 [0.94, 3.55]**
Heterogeneity: Tau² = 0.34; Chi² = 87.51, df = 2 (P < 0.00001); I² = 98%				
Test for overall effect: Z = 1.77 (P = 0.08)				
**1.1.2 VA/SCD**				
Fung 2021	-0.041	0.056	32.7%	0.96 [0.86, 1.07]
Ray WA[SSRI] 2004	-0.051	0.417	14.5%	0.95 [0.42, 2.15]
Ray WA[TCA] 2004	0.113	0.116	30.4%	1.12 [0.89, 1.41]
Whang W 2009	1.206	0.254	22.5%	3.34 [2.03, 5.49]
**Subtotal (95% CI)**			**100.0%**	**1.33 [0.88, 2.01]**
Heterogeneity: Tau² = 0.13; Chi² = 23.59, df = 3 (P < 0.0001); I² = 87%				
Test for overall effect: Z = 1.35 (P = 0.18)				

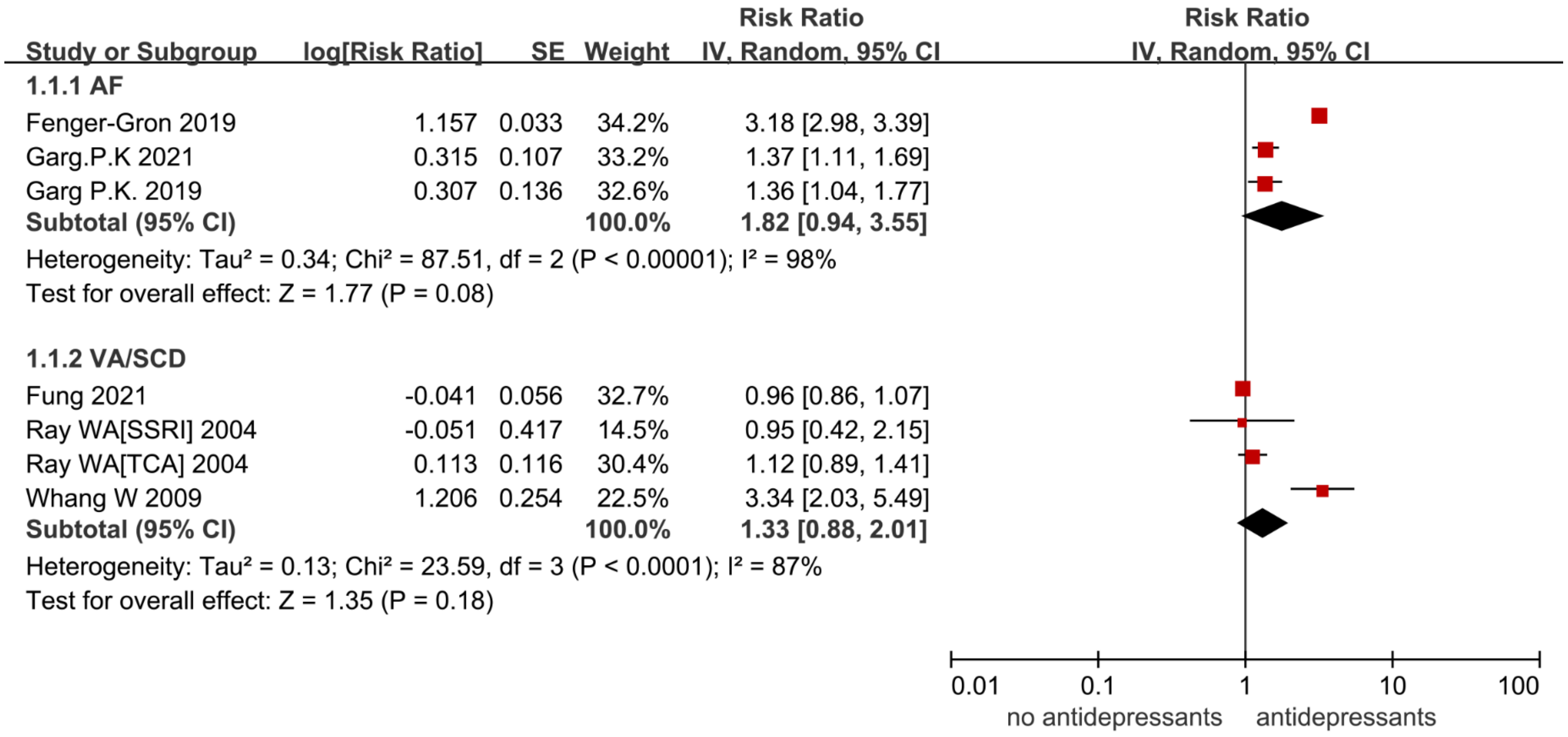

Supplementary Figure 1. Forest plot of the association between antidepressants and the risk of arrhythmia of highly heterogeneous included studies

AF=atrial fibrillation; VA= ventricular arrhythmia; SCD=sudden cardiac death; RR=risk ratio; TCA= tricyclic antidepressant; SSRI= selective serotonin reuptake inhibitor; SNRI= serotonin–norepinephrine reuptake inhibitor; CI=confidence interval; SE=standard error; IV=inverse of the variance.
